# Effect of Frying Temperatures and Times on the Quality and Flavors of Three Varieties of *Lentinus edodes*

**DOI:** 10.3390/foods14010024

**Published:** 2024-12-25

**Authors:** Yan Chen, Yaping Wang, Qinglin Guan, Xiaoli Zhou

**Affiliations:** College of Food Science and Engineering, Guiyang University, Guiyang 550005, China

**Keywords:** *Lentinus edodes*, frying conditions, nutrients, volatiles, flavor

## Abstract

The effects of frying times (1, 2, 3, and 4 min) and temperatures (140, 160, 180, and 200 °C) were investigated on the nutritional components, color, texture, and volatile compounds of three *Lentinula edodes* varieties (808, 0912, and LM) from Guizhou, China. Increased frying time and temperature significantly reduced the moisture, polysaccharide, and protein contents, while increasing hardness and chewiness, and decreasing elasticity and extrusion resilience, negatively impacting overall quality. Optimal umami and sweet amino acid retention were achieved by frying at 160 °C frying for 1–3 min or 140–180 °C for 2 min. Nine volatile compounds were identified, with sulfur-containing compound levels decreasing and ketone, aldehyde, pyrazine, and other volatile compound levels increasing as frying progressed. At temperatures above 180 °C, variety 808 displayed a duller appearance, while variety LM experienced significant water and protein loss, making them unsuitable for frying under these conditions. Conversely, variety 0912 demonstrated superior characteristics, such as retaining higher levels of aspartic acid and sulfur-containing compounds, resulting in a sweeter taste. Overall, frying for 2–3 min at 160–180 °C can preserve high nutritional quality and taste and enhance flavor characteristics relatively well. These findings provide a theoretical basis for the deep processing and utilization of *Lentinula edodes* and for standardized industrial production.

## 1. Introduction

*Lentinus edodes* (LE), commonly known as shiitake mushroom, is one of the most widely consumed edible mushrooms globally, particularly in East Asian countries, such as China, Japan, and South Korea [1]. LE is rich in essential macronutrients and micronutrients, as well as proteins, polysaccharides, amino acids, dietary fibers, minerals, vitamins, and other compounds [2,3]. These ingredients have a variety of biological activities, such as anti-tumor, anti-viral, anti-aging, antibacterial, and immunomodulatory effects [4,5,6,7,8]. Notably, LE glycoproteins demonstrate strong scavenging activity against hydroxyl- and superoxide-free radicals, enhancing its antioxidant properties [9]. Additionally, LE polysaccharides support digestive health and promote intestinal microbial fermentation [10]. Furthermore, LE reportedly reduces oxidative stress, decreases the expression of inflammatory cytokines, protects neurons, and holds potential as a treatment for Alzheimer’s disease [11]. Given these functional properties, incorporating LE into the food industry has the potential to significantly enhance the nutritional quality of various food products.

LE is mostly consumed in its cooked form, as cooking significantly alters its nutritional composition, texture, and flavor. For example, Nie et al. [12] investigated the impact of boiling times (10–120 min) on the nutritional and antioxidant properties of LE pieces and their broth, finding that different cooking conditions influenced its nutritional profile. Yao et al. [2] studied the effects of steaming, boiling, air frying, and oven baking on the volatiles of shiitake mushrooms, revealing that all cooking treatments reduced the complexity of flavor and the relative content of volatile compounds. Previous studies from our lab revealed that frying increases the proportion of sweet and umami amino acids in LE compared to other cooking methods [13].

Deep frying, one of the oldest cooking techniques, is widely used due to its practicality, efficiency, and ability to produce food in large quantities. This method not only extends the shelf life of food [14], but also imparts an appealing flavor, texture, and golden color that is deeply favored by consumers. Frying is a complex process involving a series of physical and chemical reactions. The golden-brown color of fried foods is primarily attributed to non-enzymatic browning reactions, such as Maillard and caramelization reactions, which also contribute to the unique flavors of fried products [15]. However, the sensory characteristics and underlying mechanisms of fried foods are highly complex. Safety issues, such as fat intake and acrylamide production in fried foods, have gradually attracted consumers’ attention, and the control of frying conditions is particularly important [16]. They depend on several factors, including the type and quantity of reducing sugars and amino acids in the food, as well as frying temperature and time [17]. The frying process has varying effects on the nutritional quality, taste, and bioactive compounds of foods. For instance, Poncero et al. [18] reported that frying *Agaricus bisporus*, LE, and *Pleurotus ostreatus* resulted in significant losses of proteins, minerals, and carbohydrates, alongside increased fat and energy contents. Ren et al. [19] suggested that pretreatments, such as blanching, osmotic dehydration, and coating, prior to vacuum frying were most effective in preparing vacuum-fried LE chips. Similarly, Devi et al. [20] proposed that ultrasonic and microwave-assisted vacuum frying better retained nutrients and reduced oil absorption in LE. Nevertheless, high frying temperatures are associated with several drawbacks, such as the formation of acrylamides, high oil contents, and the destruction of heat-sensitive nutrients [21]. Moreover, deep-frying conditions, such as excessive frying times or high temperatures, can cause issues, such as premature foaming, dark coloration, off-flavors, and poor frying performance [16]. Therefore, optimizing frying temperature and time is crucial to balancing the sensory quality and nutritional value of fried foods.

Despite the widespread popularity of fried foods for their golden, crispy texture and unique flavor, limited research has been conducted on how frying conditions affect LE. At present, the related research mainly focuses on the effect of cooking methods on the nutrients and antioxidant activity of LE [13] as well as the effect of pretreatment on the quality of vacuum-fried LE slices [19]. Moreover, there are few studies on the related nutrition and flavor of several Guizhou LE varieties under frying conditions (time and temperature). Thus, this study aimed to explore (1) the effects of different frying temperatures and times on the texture and color of LE, (2) the effects of different frying temperatures and times on the nutritional components of LE, and (3) the effects of different frying temperatures and times on the flavor profile of LE.

## 2. Materials and Methods

### 2.1. LE Samples

Fresh basidiomata of three varieties of LE (0912, 808, and LM) were harvested from cultivation rooms in Guizhou Province, China. Only LE samples that were free from deterioration, insect infestation, and had uniformly sized caps were selected for this study. The soil and substrate residues attached to the fresh samples were removed under gently flowing water. After cleaning, the LE samples were cut into uniform slices measuring 1 × 1 × 0.5 cm for later use.

### 2.2. Frying Processing

The three varieties of LE under different frying conditions were divided into two treatment groups: Group I included frying at 160 °C for 1, 2, 3, and 4 min, respectively; Group II included frying for 2 min at 140, 160, 180, and 200 °C, respectively. Soybean oil (Shanghai, China), purchased from a local market, was used as the frying medium. After frying, excess oil on the surface of the samples was absorbed with paper towels. The samples were then stored at −18 °C for later use. All experiments were conducted in triplicate.

### 2.3. Color Measurements

The color of fried LE samples was measured using a Hunter Lab-Eutin spectrocolorimeter (Konica Minolta, Osaka, Japan) with a 50 mm × 50 mm cuvette. The color values were expressed in terms of L* (darkness/lightness), a* (greenness/redness), and b* (blueness/yellowness). The total color difference (ΔE) was calculated using the equation: ∆E = $\sqrt{{\left(L^{*}-L_{0}\right)}^{2}+{\left(a^{*}-a_{0}\right)}^{2}+{\left(b^{*}-b_{0}\right)}^{2}}$, where L*, a*, and b* represent brightness, red–green, and yellow–blue after frying, respectively, and L_0_, a_0_, and b_0_ represent the brightness, red–green, and yellow–blue before frying, respectively. Each color value was recorded as the mean of seven measurements taken at room temperature (22–24 °C).

### 2.4. Texture Profile Analysis

Texture profile analysis was performed using a texture analyzer (TA-XT Plus, Texture Technologies, Hamilton, MA, USA) following the method reported by Nyaisaba et al. [21], with minor modifications. Fried samples were stored in separate sealed bags and placed in a desiccator to prevent moisture absorption from the environment. Samples were compressed twice to 40% of their original height using a sphere probe (P36R) at a crosshead speed of 2 mm/s. A recovery time of 5 s was set between compressions, with a trigger force of 4 G. Force–time curves obtained from the analysis included parameters, such as hardness, springiness, chewiness, and extrusion-restoring force.

### 2.5. Proximate Analysis

The chemical composition of LE (moisture, polysaccharides, and proteins) was determined in triplicate. Protein content was measured using the Nie et al. [12] method, with a conversion factor of 6.25. The moisture content was determined by heating the fresh samples at 105 °C overnight until a constant weight was achieved [19]. The polysaccharide content was analyzed using the phenol–sulfuric acid method [2], with a standard curve prepared using a standard glucose solution (Y = 1.0594x + 0.0603, R^2^ = 0.9992). All chemicals and solvents used were of analytical grade and were purchased from Sinopharm Chemical Reagent Co., Ltd. (Shanghai, China).

### 2.6. Free Amino Acid Profile

The free amino acid composition of LE was analyzed using high-performance liquid chromatography (Agilent 1100 Series, Palo Alto, CA, USA) at the School of Food Science and Technology, Jiangnan University, following the methods described by Sun et al. [22]. To prepare the samples, 1.0 g of freeze-dried LE powder was diluted with 25 mL of 5% (*v*/*v*) trichloroacetic acid and incubated for 20 min. The mixture was filtered using a double-layered filter paper and centrifuged at 2200× *g* for 30 min. The supernatant was filtered using a 0.2 μm water system filter membrane (Shanghai Xingya Purification Material Co., Shanghai, China) before analysis.

### 2.7. Volatile Compound Analysis

Volatile compounds in LE were analyzed as previously described [23], with minor modifications. For extraction using headspace solid-phase microextraction (HS-SPME), 2.0 g of sample was placed into a 20 mL headspace glass sampling vial. A DVB/CAR/PDMS SPME fiber (Supelco, Bellefonte, PA, USA) was used to extract volatile compounds at 60 °C for 30 min. The HS-SPME fiber was then inserted into the injection port of a gas chromatography–mass spectrometry (GC-MS) system for thermal desorption.

GC-MS analysis was performed using a Trace GC and a Trace MS (Finnigan Trace DSQ GC/MS, Finnigan, Waltham, MA, USA) equipped with a 3DB-624 Ultra Inert column (30 m × 250 μm × 1.4 μm, J&W Scientific, Folsom, CA, USA). The injector temperature was set to 240 °C. The initial oven temperature was maintained at 38 °C for 5 min, then increased to 140 °C at a rate of 6 °C/min, followed by a further increase to 240 °C at 10 °C/min, where it was held for 10 min. Helium was used as the carrier gas at a flow rate of 1 mL/min, with a split ratio of 1:10. Mass spectra were acquired in electron impact mode at 70 eV, with an ion source temperature of 230 °C. The mass scanning range was 25–500 u. Data were processed using a mass spectrometry system and compared with the NIST 2017 and Wiley 275 spectral libraries for identification. Volatile chemical components were quantified using the peak area normalization method.

### 2.8. Statistical Analysis

All data are presented as mean ± standard deviation. Color and texture analyses were performed by conducting seven parallel measurements, while the other experiments were conducted in triplicate. Statistical analysis was performed using SPSS 22.0 software (SPSS Inc., Chicago, IL, USA). One-way analysis of variance (ANOVA) and Duncan’s multiple comparison tests were conducted for each group, with significance set at *p* < 0.05.

## 3. Results and Discussion

### 3.1. Effect of Different Frying Conditions on the Texture and Color of LE

#### 3.1.1. Effects of Frying Conditions on the Color of LE

**Figure 1:**
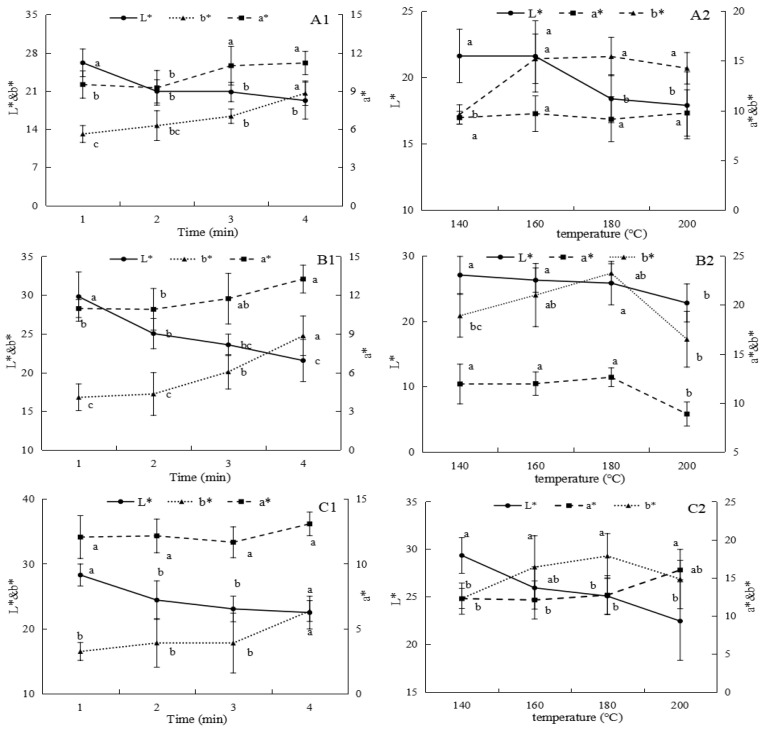
Effects of different frying conditions on the color of LE produced in Guizhou. (**A1**–**C1**) represent the color changes in LM, 808, and 0912 fried at 160 °C for different times, (**A2**–**C2**) represent the color changes in LM, 808, and 0912 fried at different temperatures for 2 min. Note: a–c: significant difference (*p* < 0.05) in different processing methods. ANOVA and Duncan test were used to analyze the significant difference among samples during different frying temperatures and times.

The color of fried food is primarily influenced by non-enzymatic browning reactions, with Maillard and caramelization reactions playing key roles. The Maillard reaction enhances desirable coloration during its second stage, whereas in the third stage, melanin formation results in progressively darker hues [24]. Therefore, appropriate frying conditions are essential for achieving visually appealing products. As shown in Figure 1, the L* values of the three varieties of LE decreased as frying temperature and time increased. This reduction in lightness is likely due to the formation of browning pigments at elevated temperatures [2], a phenomenon also observed in fried sweet potatoes [25]. Furthermore, the a* and b* values of the samples increased with the extension of frying time, indicating an enhancement in color. The loss of pigments and inactivation of polyphenol oxidase in LE may also contribute to the increase in a* and b* values [2]. However, for variety 808, the a* and b* values decreased significantly when the frying temperature exceeded 180 °C (*p* < 0.05), suggesting that this variety is unsuitable for high temperature frying.

Moreover, the surface color of the three LE varieties transitioned from golden-yellow to nearly black after frying at 160 °C for 4 min or at 200 °C for 2 min. These conditions strongly promoted brown pigment formation, resulting in darker colors. In contrast, frying at 140–180 °C for 1–3 min maintained a desirable golden-yellow appearance, making these conditions optimal for producing visually appealing fried mushroom products.

#### 3.1.2. Effects of Frying Temperature and Time on the Texture of LE

The crisp texture of fried food is closely linked to water evaporation during frying. As water escapes, a porous structure forms, allowing oil to infiltrate and interact with various components in the mushroom, creating a unique texture [15]. Frying also induces chemical reactions, such as protein denaturation, oil oxidation, amino acid degradation, and Maillard reactions, all of which contribute to the sensory properties of fried foods [24]. Table 1 and Table 2 show that as frying time and temperature increased, the moisture content of the three varieties (808, 0912, and LM) decreased. This loss of moisture resulted in increased hardness and chewiness, whereas elasticity and extrusion resilience decreased. These changes in texture could be attributed to protein denaturation, which affects the hardness and elasticity of the mushrooms. Similar adverse effects of frying time and temperature have been reported in soybean meal crisps [26]. Among the three varieties, LM retained better elasticity and extrusion resilience after frying, which may enhance its flavor. Overall, frying at 160–200 °C for 2–3 min produced fried LE with a desirable golden color and balanced texture, making it ideal for product development. However, extended frying times or excessive temperatures negatively affected texture, rendering all three LE varieties unsuitable for long-term or high-temperature frying.

### 3.2. Effects of Frying Conditions on the Nutrient Components of LE

#### 3.2.1. Effects of Frying Conditions on the Moisture Content of LE

**Figure 2:**
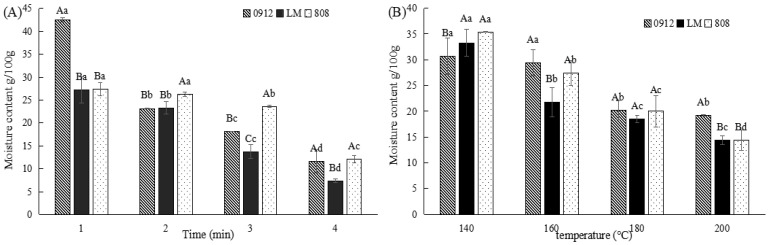
Effects of different frying conditions on the moisture content of LE produced in Guizhou. (**A**) The effect of different frying times at 160 °C on the moisture content of LE. (**B**) The effect of different frying temperatures for 2 min on the moisture content of LE. Note: A–C: significant differences (*p* < 0.05) in the three kinds of LE under the same condition; a–d: significant difference (*p* < 0.05) in different processing methods. ANOVA and Duncan test were used to analyze the significant differences among samples during different frying temperatures and times.

Moisture content plays a vital role in the quality and safety of fried foods. As shown in Figure 2, the moisture content of all three varieties (LM, 808, and 0912) decreased with increasing frying time and temperature. This decrease can be attributed to the rapid evaporation of water during frying, leading to the formation of a thicker outer shell and a reduced heat and steam transfer rate. Similar trends have been observed in salads [27] and potatoes [25]. During the early stages of frying, rapid water evaporation caused structural damage to LE cells, increasing their permeability, the outflow of cell components, and intercellular viscosity, softening the mushrooms. Prolonged frying led to further dehydration, reducing moisture content, increasing hardness, and eventually decreasing brittleness. The initial moisture contents of 0912, 808, and LM were 81.52 ± 0.09, 79.12 ± 0.47, and 80.10 ± 0.47 g/100 g, respectively. Among the varieties, 0912 exhibited a faster moisture loss in the early frying stages but stabilized after 2 min. In contrast, LM showed the highest moisture loss rate, indicating its susceptibility to over-frying. To preserve texture and prevent hardening, frying conditions should not exceed 200 °C or 2 min. Notably, when the mushrooms were fried for 4 min at 160 °C, the moisture content of all three varieties fell below 13%. Maintaining a moisture content of 13% improves storage stability and quality of dried LE, since it reduces the rate of physical and chemical changes. Therefore, frying at 160–200 °C for 2–3 min is recommended for achieving optimal sensory and moisture balance in fried LE.

#### 3.2.2. Effects of Frying Conditions on the Protein Content of LE

**Figure 3:**
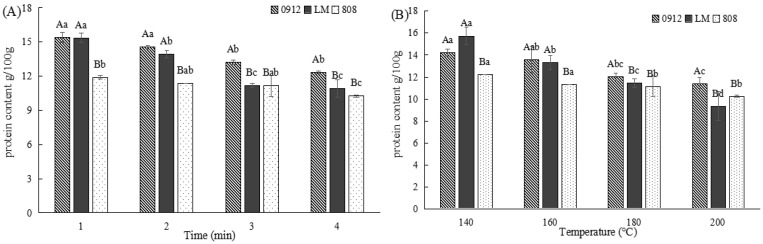
Effects of different frying conditions on the protein content of LE produced in Guizhou. (**A**) The effect of different frying times at 160 °C on the protein content of LE. (**B**) The effect of different frying temperatures for 2 min on the protein content of LE. Note: A–B: significant differences (*p* < 0.05) among the three kinds of LE under the same conditions; a–d: significant differences (*p* < 0.05) in different processing methods. ANOVA and Duncan test were used to analyze the significant differences among samples during different frying temperatures and times.

As consumer awareness grows, plant-based proteins are increasingly being recognized as high-quality protein sources. LE contains approximately 20–25% of protein [28]. However, thermal processing can alter protein structure and reduce its functional properties [12]. As shown in Figure 3, the protein content of all three varieties decreased with increasing frying time and temperature. This reduction may be due to lipid accumulation during frying, which proportionally dilutes proteins, thereby reducing the protein levels. LE exhibited a high protein loss rate, suggesting that its protein structure is less stable under high-temperature frying. Furthermore, frying temperature had a greater effect on protein content than frying time, emphasizing the importance of temperature control during the development of products using LE as a raw material.

#### 3.2.3. Effects of Frying Conditions on the Polysaccharide Content of LE

**Figure 4:**
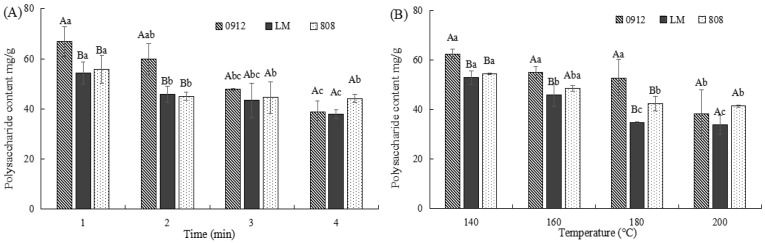
Effects of different frying conditions on the polysaccharide content of LE produced in Guizhou. (**A**) The effect of different frying times at 160 °C on the polysaccharide content of LE. (**B**) The effect of different frying temperatures for 2 min on the polysaccharide content of LE. Note: A–B: significant differences (*p* < 0.05) among the three kinds of LE under the same condition; a–c: significant differences (*p* < 0.05) among different processing methods. ANOVA and Duncan test were used to analyze the significant differences among samples during different frying temperatures and times.

Polysaccharides are a major class of bioactive compounds in LE, with antioxidant, anti-diabetic, antibacterial, anti-inflammatory, and immune-regulatory properties [5]. As shown in Figure 4, the polysaccharide content of all three varieties decreased with prolonged frying time and elevated temperature. This decrease may result from polysaccharide degradation or hindered extraction during frying. Moreover, polysaccharides may interact with nitrogen-containing substances [29], contributing to the observed decrease in L* values (brightness). Differences in polysaccharide composition and structure among the LE varieties influenced their thermal stability. For example, the polysaccharide content of variety 808 remained relatively stable after frying at 160 °C for 2 min (*p* < 0.05), indicating greater resilience to heat. In contrast, variety 0912 experienced a faster decrease in the polysaccharide content when fried at 180 °C or for longer than 3 min (*p* < 0.05). These findings suggest that controlling frying conditions is crucial for preserving polysaccharides in 0912.

#### 3.2.4. Effects of Frying Conditions on the Free Amino Acids of LE

Non-volatile compounds, such as free amino acids and nucleotides, significantly influence the taste profile of LE [30]. Free amino acids are not only key nutritional components but also react with carbohydrates and other substances during frying to form flavor compounds, such as those contributing to sweetness and a caramel-like flavor [31]. As shown in Table 3, Table 4 and Table 5, with the increase in frying time and temperature, the total amino acid content in 0912 decreased gradually, while that in LM increased first and then decreased; the total amino acid content reached the highest at 160 °C for 3 min, which may be due to the degradation of protein in the early stages of frying and the release of amino acids. The total amino acid content of 808 was the highest at 160 °C for 2–3 min, but gradually decreased with the increase in temperature, which may be related to the Maillard reaction or deamidation/dehydration reaction, which consumes amino acids [32]. A similar trend in declining free amino acid content has been reported in fried shrimp meat and fish filets [33,34]. The amino acid composition varied based on the frying conditions used. Under moderate conditions (frying for 2–3 min at 160 °C), the loss of amino acids in LE was minimal, and the content of some amino acids even increased, enhancing the flavor and aroma.

Free amino acids can be grouped into four taste categories: sweet (Ser, Gly, Thr, Ala, and Pro), fresh (Glu and Asp), bitter (His, Arg, Val, Met, Phe, and Ile), and tasteless (Lys, Tyr, and Cys-s) [30]. The sensory perception of food is closely linked to the content and proportion of these flavor-related amino acids. An analysis of amino acid ratios is shown in Table 6. The contents of glutamic acid and aspartic acid were consistently the most abundant before and after frying all three LE varieties, contributing to the umami and sweet flavor. This aligns with studies by Jiang et al. [30] who identified aspartic acid as the main contributor to the umami taste of mushrooms. Although the proportion of bitter amino acids before and after frying was also high, they likely interacted with soluble sugars, polyols, and other flavor-enhancing amino acids, balancing and masking their bitterness to create the unique taste of LE [35]. For example, Arg, while bitter, is a key compound in shrimp meat that enhances its overall flavor [33]. In summary, the proportion of flavor enhancing amino acids varied across the three LE varieties. Frying at 180 °C for 3 min was most effective in preserving the freshness and sweetness of amino acids, making it a favorable condition for developing high-quality products.

### 3.3. Effects of Frying Conditions on the Volatile Flavor of LE

**Figure 5:**
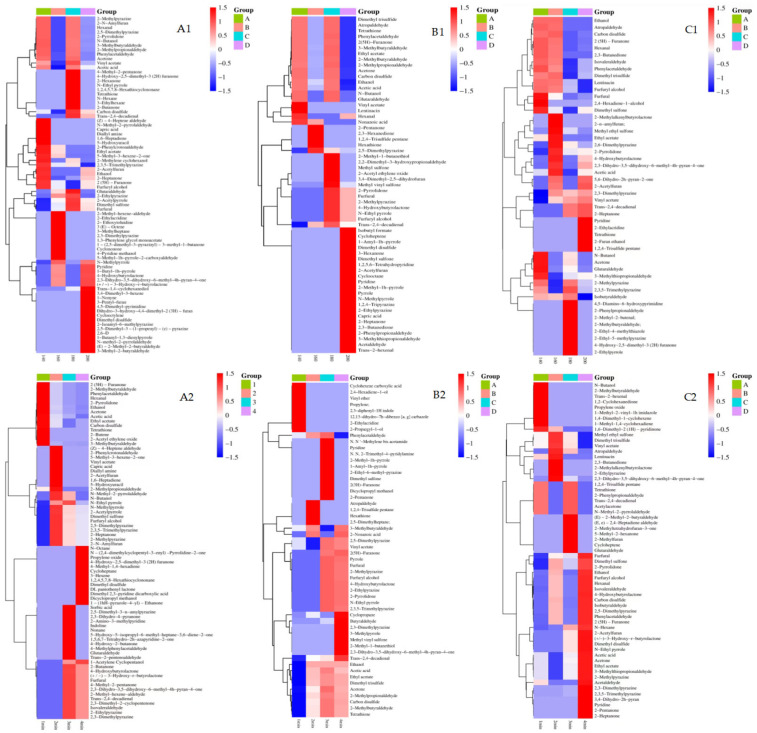
Heat map of volatile flavor compounds in different frying conditions in the three varieties of LE. Note: (**A1**–**C1**) represent the heat map of volatile components of 0912, LM, and 808 under different frying temperatures for 2 min, (**A2**–**C2**) represent the heat map of volatile components of 0912, LM, and 808 fried at 160 °C for different frying times.

Volatile compounds play an important role in the sensory evaluation of fried foods. A high temperature during frying promotes complex chemical reactions and produces unique volatile flavor components [15]. The relative proportion of these volatile compounds and the sensory threshold determine the overall flavor profile of the fried product. As shown in Figure 5, LE contains a variety of volatile compounds, including alcohols, aldehydes, ketones, acids, esters, pyrazines, pyrroles, and sulfur-containing compounds. The unique odor of the alcohols gives different mushrooms unique aromas, including fruity, vegetable, flowery, fatty, and roasted flavors [30]. Under different frying conditions, the volatile components with higher content in the three varieties of LE mainly included aldehydes and sulfur-containing compounds. As an important part of mushroom flavor substances, aldehydes can confer different flavors, such as fruity, vegetable, baking, and caramel flavors [35,36]. The results showed that the proportion of aldehydes in the three varieties of LE under different frying conditions was in the range of 11.89–39.97%, and the content of aldehydes in 0912 was as high as 32.21% at 160 °C 1 min. The aldehyde content of 808 was 39.97% at 160 °C for 3 min. The maximum aldehyde content of LM was 39.08% at 180 °C for 2 min. Sulfur compounds are formed by dithioalkane intermediates produced by the polymerization of lentinic acid through glutamate transpeptidase activity, affecting the overall odor of the mushroom [30]. The sulfur compound content of LM and 808 reached a maximum of 53.64% and 53.05%, respectively, when fried at 160 °C for 2 min; thereafter, it gradually decreased with increasing temperature and time. Overall, the total volatile compound content of the three LE varieties exhibited fluctuations.

Heat maps were used to visualize the distribution of flavor compounds under different frying conditions (Figure 5). Cluster analysis revealed clear differences in the volatile profiles of the three LE varieties across frying times and temperatures. For example, pyrazine and pyrrole compound levels increased significantly in 0912, with the 2-pentylfuran level remaining relatively stable. This produced a sweet and caramel-like flavor, consistent with the findings for furan compounds in fried chicken breast at higher temperature [37]. LM uniquely produced 2 (5H)-furanone and 2-pyrrolidone, whereas 808 exhibited distinct compounds, such as 2-heptanone and additional aldehydes. These findings suggest that higher temperatures and longer frying times promote the cracking and oxidation of oils, forming heterocyclic ketones and lactones, and increasing the content of aldehydes, pyrazines, pyrroles, and other substances [38]. However, certain volatile substances were only detected at moderate conditions (2–3 min or 160–180 °C), suggesting that they may represent intermediate flavor compounds in LE. In summary, frying at 160–180 °C for 2–3 min yielded the most balanced proportion of volatile compounds (pyrazine and pyrrole), producing optimal flavor profiles across all three varieties. This makes these conditions ideal for product development.

## 4. Conclusions

In this study, we investigated the nutritional, color, texture, and volatile components of three kinds of LE (808, 0912, and LM) in Guizhou province under different frying times and temperature conditions. Increasing the frying time and temperature significantly decreased the moisture, polysaccharide, and protein contents of the three kinds of LE, while the hardness and chewiness increased gradually. A frying temperature of 160 °C or in the rage of 140–180 °C for 1–3 min led to the preservation of sweet amino acids in LE. Specifically, frying for 2–3 min at 160–180 °C yielded LE with a golden yellow surface, with moderate hardness and the best flavor and texture. When the frying temperature was higher than 180 °C, the a* and b* values of 808 decreased, resulting in a dim color. Notably, the moisture and protein loss of LM was larger, while the nutritional value of 0912 was higher, and its high content of aspartic acid and sulfur compounds gave it a unique sweet taste. In addition, the proportions of the three LE volatile compounds showed a positive correlation with frying time and temperature. Controlling frying time and temperature can enhance the aroma potential. In summary, this study provided a theoretical basis for the development of fried mushrooms and promotion of the efficient use of mushroom resources and industrial development.

## Figures and Tables

**Table 1:** Effects of different frying times at 160 °C on the texture profile of LE produced in Guizhou.

Texture Properties	Varieties	Frying Time (min)			
		1	2	3	4
Hardness (N)	0912	818.97 ± 107.74 ^c^	1056.13 ± 131.73 ^bc^	1178.47 ± 32.27 ^ab^	1395.14 ± 87.20 ^a^
	LM	824.89 ± 93.02 ^b^	826.18 ± 187.92 ^b^	1280.67 ± 176.77 ^a^	1334.52 ± 189.18 ^a^
	808	840.76 ± 125.65 ^c^	996.29 ± 51.25 ^bc^	1255.22 ± 49.23 ^ab^	1457.41 ± 310.06 ^a^
Chewiness (mJ)	0912	820.05 ± 20.76 ^b^	862.98 ± 87.03 ^b^	972.99 ± 86.14 ^b^	1279.43 ± 215.80 ^a^
	LM	560.04 ± 79.29 ^b^	636.87 ± 101.53 ^b^	857.54 ± 53.27 ^a^	942.45 ± 50.94 ^a^
	808	740.83 ± 144.84 ^c^	995.99 ± 94.22 ^b^	1141.15 ± 147.14 ^b^	1131.25 ± 20.59 ^a^
Springiness (%)	0912	0.87 ± 0.12 ^a^	0.81 ± 0.10 ^ab^	0.77 ± 0.03 ^ab^	0.75 ± 0.04 ^b^
	LM	0.93 ± 0.03 ^a^	0.89 ± 0.07 ^ab^	0.83 ± 0.05 ^bc^	0.75 ± 0.04 ^c^
	808	0.91 ± 0.06 ^a^	0.87 ± 0.10 ^a^	0.82 ± 0.05 ^b^	0.83 ± 0.11 ^b^
Extrusion-restoring force (mm)	0912	0.37 ± 0.04 ^a^	0.31 ± 0.05 ^a^	0.24 ± 0.05 ^b^	0.19 ± 0.03 ^b^
	LM	0.39 ± 0.06 ^a^	0.32 ± 0.04 ^bc^	0.36 ± 0.07 ^ab^	0.27 ± 0.05 ^bc^
	808	0.37 ± 0.04 ^a^	0.31 ± 0.05 ^a^	0.24 ± 0.05 ^b^	0.19 ± 0.03 ^b^

^a–c^ significant differences (*p* < 0.05) in different processing methods. Analysis of variance (ANOVA) and Duncan’s test were used to analyze significant differences among the samples at different frying times.

**Table 2:** Effects of different frying temperatures for 2 min on the texture characteristics of LE produced in Guizhou.

Texture Properties	Varieties	Frying Temperature (°C)			
		140	160	180	200
Hardness (N)	0912	885.75 ± 40.14 ^c^	1025.76 ± 75.61 ^bc^	1198.88 ± 90.68 ^ab^	1325.26 ± 238.70 ^a^
	LM	724.66 ± 29.64 ^b^	858.67 ± 93.74 ^b^	1193.40 ± 97.71 ^a^	1654.95 ± 97.35 ^a^
	808	840.76 ± 125.5 ^c^	996.29 ± 51.25 ^bc^	1255.22 ± 49.23 ^ab^	1457.41 ± 310.06 ^a^
Chewiness (mJ)	0912	867.89 ± 92.22 ^b^	872.64 ± 102.77 ^b^	932.94 ± 82.44 ^b^	1268.63 ± 56.80 ^a^
	LM	639.58 ± 52.98 ^b^	627.46 ± 63.74 ^b^	740.78 ± 95.58 ^b^	1068.30 ± 23.22 ^a^
	808	546.71 ± 99.22 ^b^	572.57 ± 62.45 ^b^	669.14 ± 91.88 ^b^	1064.73 ± 203.27 ^a^
Springiness (%)	0912	0.91 ± 0.04 ^a^	0.85 ± 0.05 ^ab^	0.81 ± 0.04 ^ab^	0.77 ± 0.09 ^b^
	LM	0.91 ± 0.06 ^a^	0.87 ± 0.10 ^a^	0.82 ± 0.05 ^b^	0.83 ± 0.11 ^b^
	808	0.85 ± 0.08 ^a^	0.82 ± 0.11 ^a^	0.85 ± 0.08 ^a^	0.78 ± 0.06 ^b^
Extrusion-restoring force (mm)	0912	0.36 ± 0.03 ^a^	0.32 ± 0.02 ^b^	0.27 ± 0.03 ^c^	0.26 ± 0.04 ^c^
	LM	0.37 ± 0.04 ^a^	0.32 ± 0.06 ^ab^	0.32 ± 0.03 ^ab^	0.28 ± 0.08 ^b^
	808	0.36 ± 0.07 ^a^	0.31 ± 0.02 ^a^	0.23 ± 0.06 ^b^	0.22 ± 0.05 ^b^

^a–c^ significant differences (*p* < 0.05) in different processing methods. Analysis of variance (ANOVA) and Duncan’s test were used to analyze significant differences among the samples at different frying temperatures.

**Table 3:** Effects of different frying conditions on the free amino acids of 0912 produced in Guizhou.

Amino Acids	Frying Conditions							
	Time (min) at 160 °C				Temperature (°C) for 2 min			
	1	2	3	4	140	160	180	200
Asp	0.85	0.83	0.79	0.72	0.89	0.88	0.84	0.53
Glu	2.23	1.93	1.89	1.30	0.97	1.34	0.91	0.63
Ser	0.03	0.03	0.03	0.03	0.03	0.02	0.02	0.02
Gly	0.22	0.17	0.23	0.15	0.21	0.20	0.19	0.12
Thr	0.50	0.43	0.55	0.35	0.66	0.65	0.61	0.38
Ala	0.79	0.69	0.74	0.57	1.11	0.74	1.05	0.70
Pro	0.17	0.14	0.22	0.16	0.24	0.23	0.40	0.17
His	0.24	0.23	0.24	0.18	0.23	0.22	0.19	0.12
Arg	1.00	0.75	0.81	0.65	0.62	0.62	0.52	0.37
Val	0.59	0.53	0.62	0.41	0.75	0.73	0.69	0.44
Met	0.45	0.37	0.41	0.28	0.86	0.85	0.78	0.30
Phe	0.22	0.20	0.23	0.14	0.34	0.33	0.30	0.18
Ile	0.23	0.21	0.25	0.16	0.28	0.27	0.26	0.15
Leu	0.30	0.27	0.31	0.19	0.40	0.38	0.36	0.20
Tyr	0.15	0.14	0.13	0.11	0.11	0.12	0.11	0.07
Cys	0.01	0.02	0.02	0.01	0.02	0.03	0.02	0.01
Lys	0.43	0.37	0.41	0.26	0.52	0.49	0.42	0.27
Total	8.41	7.31	7.88	5.67	8.24	8.1	7.67	4.66

**Table 4:** Effects of different frying conditions on the free amino acids of LM produced in Guizhou.

Amino Acids	Frying Condition							
	Time (min) at 160 °C				Temperature (°C) for 2 min			
	1	2	3	4	140	160	180	200
Asp	0.45	0.72	0.53	0.49	0.48	0.42	0.73	0.47
Glu	0.42	0.75	1.07	0.72	0.74	0.50	0.99	0.72
Ser	0.06	0.08	0.14	0.11	0.09	0.10	0.10	0.09
Gly	0.05	0.07	0.19	0.14	0.13	0.11	0.17	0.12
Thr	0.14	0.23	0.44	0.30	0.23	0.24	0.37	0.26
Ala	0.12	0.22	0.60	0.48	0.32	0.36	0.57	0.40
Pro	0.05	0.10	0.43	0.17	0.15	0.12	0.16	0.13
His	0.12	0.11	0.18	0.15	0.15	0.14	0.15	0.13
Arg	0.32	0.59	0.54	0.44	0.37	0.39	0.67	0.57
Val	0.17	0.19	0.55	0.41	0.30	0.31	0.50	0.35
Met	0.28	0.08	0.56	0.44	0.49	0.39	0.52	0.35
Phe	0.02	0.05	0.19	0.12	0.06	0.08	0.17	0.10
Ile	0.01	0.04	0.22	0.14	0.06	0.08	0.17	0.11
Leu	0.02	0.05	0.29	0.19	0.09	0.10	0.23	0.15
Tyr	0.16	0.15	0.22	0.21	0.19	0.17	0.17	0.15
Cys	0.01	0.03	0.01	0.03	0.01	0.05	0.01	0.01
Lys	0.08	0.21	0.35	0.23	0.14	0.19	0.32	0.22
Total	2.48	3.67	6.51	4.77	4.00	3.75	6.00	4.33

**Table 5:** Effects of different frying conditions on the free amino acids of 808 produced in Guizhou.

Amino Acids	Frying Condition							
	Time (min) at 160 °C				Temperature (°C) for 2 min			
	1	2	3	4	140	160	180	200
Asp	0.75	0.82	0.75	0.51	0.83	0.77	0.17	0.68
Glu	1.72	2.10	2.17	1.49	2.30	1.74	1.25	1.03
Ser	0.13	0.14	0.15	0.11	0.14	0.12	0.09	0.03
Gly	0.18	0.23	0.20	0.15	0.22	0.18	0.17	0.09
Thr	0.40	0.46	0.45	0.32	0.47	0.36	0.33	0.21
Ala	0.71	0.80	0.72	0.65	0.77	0.72	0.55	0.43
Pro	0.18	0.18	0.24	0.18	0.16	0.24	0.13	0.16
His	0.22	0.20	0.25	0.20	0.24	0.17	0.09	0.13
Arg	0.76	0.99	0.81	0.74	0.92	0.96	0.43	0.75
Val	0.51	0.61	0.57	0.41	0.61	0.49	0.42	0.29
Met	0.37	0.39	0.41	0.36	0.48	0.29	0.29	0.19
Phe	0.21	0.26	0.27	0.14	0.29	0.19	0.17	0.12
Ile	0.22	0.28	0.27	0.15	0.28	0.22	0.18	0.13
Leu	0.31	0.39	0.39	0.20	0.41	0.29	0.24	0.18
Tyr	0.18	0.19	0.20	0.16	0.20	0.18	0.31	0.08
Cys	0.01	0.01	0.01	0.01	0.04	0.02	0.02	0.02
Lys	0.31	0.33	0.34	0.23	0.43	0.25	0.27	0.17
Total	7.17	8.38	8.20	6.01	8.79	7.19	5.11	4.69

**Table 6:** Analysis of the proportion of flavoring amino acids in the three varieties of LE under different frying conditions (%).

Flavor Characteristics	Varieties	Frying Condition							
		Time (min) at 160 °C				Temperature (°C) for 2 min			
		1	2	3	4	140	160	180	200
umami	0912	36.58	37.71	33.97	35.66	21.55	21.60	21.95	22.95
	LM	34.90	40.01	24.53	25.37	30.41	24.46	28.70	27.55
	808	34.34	34.92	35.64	33.28	35.58	34.97	27.74	36.60
sweet	0912	20.28	20.02	22.42	22.22	26.01	25.48	28.54	27.58
	LM	17.40	19.43	27.57	25.15	22.91	24.88	22.85	23.04
	808	22.36	21.57	21.46	23.45	19.97	22.51	24.91	19.46
bitter	0912	36.10	35.07	36.57	35.40	44.92	45.28	42.59	42.49
	LM	37.78	29.89	38.97	39.69	38.17	39.59	40.08	40.60
	808	36.27	37.17	36.20	36.57	36.83	36.31	35.68	38.07
tasteless	0912	7.03	7.20	7.04	6.72	7.52	7.64	6.92	6.98
	LM	9.91	10.67	8.94	9.79	8.51	11.06	8.36	8.80
	808	7.03	6.34	6.70	6.71	7.63	6.21	11.66	5.87

## Data Availability

The original contributions presented in the study are included in the article, further inquiries can be directed to the corresponding author.

## References

[1] Xu X., Yan H., Tang J., Chen J., Zhang X. (2014). Polysaccharides in Lentinus edodes: Isolation, structure, immunomodulating activity and future prospective. Crit. Rev. Food Sci. Nutr..

[2] Yao F., Gao H., Yin C.M., Shi D.F., Fan X.Z. (2013). Effect of Different cooking methods on the bioactive components, color, texture, microstructure, and volatiles of shiitake mushrooms. Foods.

[3] He Y., Lai H., Liang J., Cheng L., He L., Wang H., Teng Q., Cai W., Wang R., Zhu L. (2024). Optimization co-culture of monascus purpureus and saccharomyces cerevisiae on selenium-enriched lentinus edodes for increased monacolink production. J. Fungi.

[4] Gyun S.P., Juhyun S., Seongwoo H., Judy G., Jae-Wook O. (2024). Anticarcinogenic potential of the mushroom polysaccharide lentinan on gastric and colon cancer cells: Antiproliferative, antitumorigenic, Mu-2-related death-inducing gene, MUDENG ramifications. J. Ind. Eng. Chem..

[5] Sheng K., Wang C., Chen B., Kang M., Wang M., Liu K., Wang M. (2021). Recent advances in polysaccharides from Lentinus edodes (Berk.): Isolation, structures and bioactivities. Food Chem..

[6] Luo J., Ganesan K., Xu B. (2024). Unlocking the power: New insights into the anti-aging properties of mushrooms. J. Fungi.

[7] Hu X., Cheng D., Zhang Y., Li P., Wu X., Fu J. (2024). Fermented Lentinus edodes extract containing α-glucan ameliorates concanavalin A-induced autoimmune hepatitis in mice. Food Sci. Hum. Wellness.

[8] Sayari M., Pradeep S.N. (2024). Extraction of chitin-glucan complex from shiitake (*Lentinula edodes*) fruiting bodies using natural deep eutectic solvents and its prebiotic potential. Int. J. Biol. Macromol..

[9] Gao J., Li X., Jia S., Zeng H., Zheng B. (2023). Structural characterization and antioxidant activity of a glycoprotein isolated from shiitake mushrooms. Food Biosci..

[10] Zhao F., Li M., Luo M., Zhang M., Yuan Y.H., Niu H.L., Yue T. (2024). The dose-dependent mechanism behind the protective effect of lentinan against acute alcoholic liver injury via proliferating intestinal probiotics. Food Funct..

[11] Xin Y., Fang F., Yue Q., Luo Y., Tian S., Cheng L.H., Wang X.C., Yang X.L., Luo L., Meng F.L. (2024). Microenvironment modulating nanogels by Shiitake-derived lentinan and a reactive oxygen species scavenging conjugated polymer for the treatment of Alzheimer’s disease. Nano Today.

[12] Nie Y., Yu M., Zhou H., Zhang P., Yang W., Li B. (2020). Effect of boiling time on nutritional characteristics and antioxidant activities of Lentinus edodes and its broth. CyTA J. Food.

[13] Zhou X.L., Guan Q.L., Wang Y.L., Lin D., Du B. (2022). Effect of different cooking methods onnutrients, antioxidant activities and flavors of three varieties of Lentinus edodes. Foods.

[14] Van Boekel M., Fogliano V., Pellegrini N., Stanton C., Scholz G., Lalljie S., Somoza V., Knorr D., Jasti P.R., Eisenbrand G. (2010). Areview on the beneficial aspects of food processing. Mol. Nutr. Food Res..

[15] Chang C., Wu G., Zhang H., Jin Q., Wang X. (2019). Deep-fried flavor: Characteristics, formation mechanisms, and influencing factors. Crit. Rev. Food Sci. Nutr..

[16] Asokapandian S., Swamy G.J., Hajjul H. (2019). Deep fat frying of foods: A critical review on process and product parameters. Crit. Rev. Food Sci. Nutr..

[17] Zhang Q., Saleh A.S., Chen J., Shen Q. (2012). Chemical alterations taken place during deep-fat frying based on certain reaction products: A review. Chem. Phys. Lipids.

[18] Roncero-Ramos I., Mendiola-Lanao M., Pérez-Clavijo M., Delgado-Andrade C. (2016). Effect of different cooking methods on nutritional value and antioxidant activity of cultivated mushrooms. Int. J. Food Sci. Nutr..

[19] Ren A., Pan S., Li W., Chen G., Duan X. (2018). Effect of various pretreatments on quality attributes of vacuum-fried shiitake mushroom chips. J. Food Qual..

[20] Devi S., Zhang M., Law C. (2018). Effect of ultrasound and microwave assisted vacuum frying on mushroom (*Agaricus bisporus*) chips quality. Food Biosci..

[21] Nyaisaba B.M., Miao W., Hatab S., Siloam A., Chen M., Deng S. (2019). Effects of cold atmospheric plasma on squid proteases and gel properties of protein concentrate from squid (*Argentinus ilex*) mantle. Food Chem..

[22] Sun Y.J., Lv F.Y., Tian J.H., Ye X.Q., Chen J.C., Sun P.L. (2019). Domestic cooking methods affect nutrient, phytochemicals, and flavor content in mushroom soup. Food Sci. Nutr..

[23] Li W., Wang J., Chen W., Yang Y., Zhang J., Feng J., Yu H., Li Q. (2019). Analysis of volatile compounds of Lentinula edodes grown in different culture substrate formulations. Food Res. Int..

[24] Wang X., Mcclements D.J., Xu Z., Meng M., Qiu C., Long J., Jin Z., Chen L. (2023). Recent advances in the optimization of the sensory attributes of fried foods: Appearance, flavor, and texture. Trends Food Sci. Technol..

[25] Abd Rahman N.A., Abdul Razak S., Lokmanalhakim L., Taip F., Mustapa Kamal S. (2017). Response surface optimization for hot air-frying technique and its effects on the quality of sweet potato snack. Food Process Eng..

[26] AL Jumayi H.A., Darwish A.M.G. (2021). Frying time and temperature conditions’ influences on physicochemical, texture, and sensorial quality parameters of barley-soybean chips. J. Food Qual..

[27] Fikry M., Khalifa I., Sami R., Khojah E., Ismail K.A., Dabbour M. (2021). Optimization of the frying temperature and Time for preparation of healthy falafel using air frying technology. Foods.

[28] Fakhreddin S. (2019). Characterization of different mushrooms powder and its application in bakery products: A review. Int. J. Food Prop..

[29] Hwang I.S., Chon S.Y., Bang W.S., Kim M.K. (2021). Influence of roasting temperatures on the antioxidant properties, β-Glucan content, and volatile flavor profiles of shiitake mushroom. Foods.

[30] Jiang C., Duan X., Lin L., Wu W., Li X., Zeng Z., Luo Q., Liu Y. (2023). A review on the edible mushroom as a source of special flavor: Flavor categories, influencing factors, and challenges. Food Front..

[31] Luo D., Wu J., Ma Z., Tang P., Liao X., Lao F. (2020). Production of high sensory quality Shiitake mushroom (*Lentinus edodes*) by pulsed air-impingement jet drying (AID) technique. Food Chem..

[32] Souza V.G.L., Pires J.R.A., Vieira É.T., Coelhoso I.M., Duarte M.P., Fernando A.L. (2018). Shelf life assessment of fresh poultry meat packaged in novel bionanocomposite of chitosan/montmorillonite incorporated with ginger essential oil. Coatings.

[33] Zhou M., Shi G., Deng Y., Wang C., Qiao Y., Xiong G., Wang L., Wu W., Shi L., Ding A. (2022). Study on the physicochemical and flavor characteristics of air frying and deep frying shrimp (crayfish) meat. Front. Nutr..

[34] Oluwaniyi O.O., Dosumu O.O., Awolola G.V. (2010). Effect of local processing methods (boiling, frying and roasting) on the amino acid composition of four marine fishes commonly consumed in Nigeria. Food Chem..

[35] Hou H., Liu C., Lu X.S., Fang D.L., Hu Q.H., Zhang Y.Y., Zhao L.Y. (2021). Characterization of flavor frame in shiitake mushrooms (*Lentinula edodes*) detected byHS-GC-IMS coupledwith electronic tongue and sensory analysis: Influence of drying techniques. LWT Food Sci. Technol..

[36] Zhuang J., Xiao Q., Feng T., Huang Q., Ho C., Song S. (2020). Comparative flavor profile analysis of four different varieties of Boletus mushrooms by instrumental and sensory techniques. Food Res. Int..

[37] Noh E., Yim J., Lee K.G. (2023). Analysis of volatile compounds and 5-hydroxymethylfurfural in fried chicken breast produced by air and deep-fat frying. Food Sci. Biotechnol..

[38] Benet I., Guàrdia M.D., Ibañez C., Solà J., Arnau J., Roura E. (2016). Low intramuscular fat (but high in PUFA) content in cooked cured pork ham decreased Maillard reaction volatiles and pleasing aroma attributes. Food Chem..

